# Whole-Exome Sequencing Identifies a Novel *CPT2* Mutation in a Pedigree With Gout

**DOI:** 10.3389/fcell.2022.802635

**Published:** 2022-03-16

**Authors:** Yong Guo, Jing Jin, Zhenni Zhou, Yihui Chen, Li Sun, Chunwu Zhang, Xiaoru Xia

**Affiliations:** ^1^ Department of Urology, The First Affiliated Hospital of Wenzhou Medical University, Wenzhou, China; ^2^ Zhejiang Center for Clinical Laboratory, Zhejiang Provincial People’s Hospital, Affiliated People’s Hospital, Hangzhou Medical College, Hangzhou, China; ^3^ Department of Internal Medicine, Yueqing People’s Hospital, Yueqing, Wenzhou, China; ^4^ Wenzhou Medical University, Wenzhou, China; ^5^ Department of Rheumatology, The First Affiliated Hospital of Wenzhou Medical University, Wenzhou, China; ^6^ Department of Injury Orthopaedics, The First Affiliated Hospital of Wenzhou Medical University, Wenzhou, China

**Keywords:** whole-exome sequencing, novel mutation, gout, CPT2 gene, pedigree

## Abstract

**Background:** Gout is a common inflammatory arthritis, and its exact pathogenesis remains unclear. Multiple studies have demonstrated that genetic factors play important roles in the development of gout. This study aims to investigate the genetic basis of gout in a three-generation pedigree of affected individuals.

**Methods:** Whole-exome sequencing (WES), comprehensive variant analyses, and co-segregation testing were performed. The effects of candidate variants on protein localization and cellular expression were analyzed, as were interactions with gout-related genes.

**Results:** After comprehensive bioinformatic analysis, Sanger sequencing validation, and pedigree co-segregation analysis, we identified a rare heterozygous missense variant (c.1891C > T, p.R631C) in *CPT2*. Although no associated changes in localization were observed, the fluorescence intensity of p.R631C mutants was obviously reduced in comparison to the wild-type protein, suggesting that protein degradation is induced by the mutant. Furthermore, our results also indicate that the c.1891C > T variant influences the ability of CPT2 to bind UCP2.

**Conclusion:** This study identified a rare *CPT2* mutation in a large Chinese pedigree with gout. Functional studies were used to define the effect of this mutant. This study provides novel insight into the genetic etiology of gout.

## Introduction

Gout is a common inflammatory arthritis caused by the deposition of monosodium urate crystals in and around the joints following longstanding hyperuricaemia (Richette and Bardin, 2010). As the aging population grows, the global burden of gout continues to rise. Although the exact pathogenesis of gout remains unclear, rapid expansion in our knowledge of genetic factors over recent years has expanded our understanding of its etiology (Major et al., 2018). Evidence from genome-wide association studies (GWAS) has demonstrated important roles for a genetic basis of gout. Multiple genetic loci have been reported to be associated with gout, including *ABCG2*, *ALDH16A1*, *ATXN2*, *BCAS3*, *CUX2*, *GCKR*, *KCNQ1*, *PDZK1*, *RFX3*, and *SLC2A9* (Sulem et al., 2011; Köttgen et al., 2013; Li et al., 2015a; Matsuo et al., 2016; Phipps-Green et al., 2016; Nakayama et al., 2017). Of note, previous GWAS studies were insufficient to report associations between rare variants and gout, due to methodological limitations.

Whole-exome sequencing (WES) has proven to be a powerful tool for discovering rare mutations, and this approach has been successfully applied to a wide range of disorders (Huang et al., 2018; Huang et al., 2019). However, only a few reports have been made concerning patients with gout. Recently, our group performed WES in a pedigree with early-onset gout (Huang et al., 2020). After comprehensive variant analyses and co-segregation testing, a novel missense variant (c.277C > A, p.L93M) in *SLC16A9* was identified (Huang et al., 2020). This study indicates that WES can be used to provide new insight into the genetic etiology of gout.

The *CPT2* gene encodes carnitine palmitoyltransferase II, an enzyme that participates in fatty acid oxidation. Together with carnitine palmitoyltransferase I, the encoded protein oxidizes long-chain fatty acids in the mitochondria. Defects in this gene are associated with mitochondrial long-chain fatty-acid (LCFA) oxidation disorders, known as CPT II deficiency (Bonnefont et al., 1996). However, the association between *CPT2* gene and gout has never been reported.

In this study, we aimed to investigate the genetic basis of gout in a three-generation affected pedigree. Ultimately, we identified a rare missense variant in the *CPT2* gene (c.1891C > T, p.R631C). Evidence from further functional experiments supports a role for this mutation in the pathogenesis of gout.

## Materials and Methods

### Participant Recruitment

This study was approved by the Ethics Committee of the First Affiliated Hospital of Wenzhou Medical University (Wenzhou, China; approval no. 2018-020). All participants were administered according to the principles of the Declaration of Helsinki. All subjects provided informed consent. Diagnosis of gout was performed by physicians in accordance with the 2015 gout classification criteria defined by the American College of Rheumatology/European League Against Rheumatism Collaborative Initiative (Neogi et al., 20152015). Patients with inherited metabolic disorders, including glycogen storage diseases and Lesch-Nyhan syndrome, were excluded in this study. Patient information is provided in Table 1. Patients reported suffering monoarthritis in the first metatarsophalangeal joint (MTP1), ankles, or knees, which was relieved within 1 week.

**TABLE 1:** Clinical information and genotypes

Individual ID	Gender	Age	Genotypes	Uric acid (mg/L)	Hyperuricemia (+/−)	Arthritis	Tophi	Comorbidity				
								Hyper tension	Hyper lipermia	Hyper glycemia	Obesity (BMI)	Hyper bilirubinaemia
II:1	M	67	+/−	617	+	+	+	+	-	-	+	+
II:2	F	62	−/−	347	-	-	-	+	+	+	+	N/A
II:3	M	64	+/−	474	+	+	-	+	-	-	+	-
II:4	F	60	−/−	N/A	-	-	-	-	N/A	N/A	-	N/A
II:5	M	57	+/−	472	+/−	-	-	+	-	+	+	-
II:7	M	51	+/−	580	+	+	+	+	-	-	+	+
II:8	F	47	−/−	350	-	-	-	-	N/A	-	+	N/A
II:11	F	68	−/−	364	-	-	-	-	-	-	+	-
III:3	F	36	+/−	451	+	+	-	-	-	-	-	-
III:5	M	25	−/−	378	-	-	-	-	-	-	-	-
III:6	M	34	+/−	610	+	+	-	-	-	-	+	-
III:9	M	33	−/−	325	-	-	-	-	-	-	-	-
III:11	M	45	−/−	249	-	-	-	-	-	-	-	+

### Whole Exome Sequencing and Variant Calling

Genomic DNA was isolated from peripheral blood according to standard procedures using a Qiamp Blood DNA mini Kit (Qiagen, Hilden, Germany). About 2 µg of genomic DNA sample was randomly sheared to 200–250 bp in size using a Covaris S220 System, after which the fragments of DNA were ligated to attach A-tails and adapters to both ends. After amplification, purification, and hybridization of adapter-ligated DNA, as well as the removal of nonhybridized fragments, capture and enrichment of exome regions were conducted using an Agilent SureSelect Human All Exon V6 kit (Agilent Technologies, Palo Alto, CA, United States) according to the manufacturer’s protocol. High-throughput library sequencing was performed on an Illumina HiSeq4000 Analyzer (Illumina, San Diego, CA, United States).

Raw sequencing data obtained from the sequencer was processed according to a customized bioinformatics pipeline as previously described (Liu et al., 2018). Briefly, raw reads were filtered to remove sequence adapters and low-quality reads based on Phred-scaled quality scores <30 and read lengths <80 bp using FastQC software (version 1.11.4). After quality control testing, remaining reads were aligned to the human reference genome (GRCh37/hg19) using the Burrows-Wheeler alignment tool (Li and Durbin, 2010) and further visualized using the SplicingViewer software (Liu et al., 2012). The Genome Analysis ToolKit (GATK; version 4.0.10.0) was used to remove duplicated reads, perform local realignment, and recalibrate map quality scores. SNV and Indel variant calling were performed using GATK Unified Genotype (version 4.0.10.0).

### Variant Annotation and Prioritization

All variants were functionally annotated as previously described (Li et al., 2015b) using mirTrios with integrated ANNOVAR. Variants were then prioritized by allele frequency, evolutionary conservation, and predicted effect on protein function, as well as based on segregation testing for disease phenotype. Variants were discarded if the base on allele frequency was present at more than 0.01% in any publicly available databases (Chen et al., 2019), including Genome Aggregation Database (http://gnomad.broadinstitute.org/), Exome Aggregation Consortium (ExAC, http://exac.broadinstitute.org/), 1,000 Genome (http://www.1000genomes.org), and NHLBI Exome Sequencing Project (ESP, http://evs.gs.washington.edu/EVS/). The effects of detected variants were assessed by four *in silico* prediction programs: SIFT, Polyphen-2, CADD, and MutationTaster. Variants were considered pathogenic if they were predicted to be deleterious or damaging by all prediction tools. Sanger sequencing (primers: CPT2-5F, GCT​TTG​ACC​GAC​ACT​TGT​TTG; CPT2-5R, TGG​TCT​CAA​ACT​CCT​GAC​CT) was used to confirm family screening of identified candidates and segregation testing.

### Expression Vector Construction and Mutagenesis

The full-length coding sequences of wild-type CPT2 and UCP2 were synthesized *in vitro* by Jiangxi Zhonghong Boyuan Biological Technology Company and cloned into the pcDNA3.1 vector. Site-directed mutagenesis was performed on wild-type *CPT2* to create the c.1891C > T variant in the pcDNA3.1 vector using specific primers according to the standard procedures of the KOD-Plus-Mutagenesis Kit (Toyobo Life Science). Green fluorescent protein (GFP) and a His tag were then fused to the N terminus of wild-type *CPT2* in the pcDNA3.1 vector, via dual-enzyme digestion with HindIII and NotI (New England Biolabs). Similarly, red fluorescent protein (RFP) and a His tag were fused to the N terminus of mutant *CPT2* mutant in the pcDNA3.1 vector using the same dual-enzyme digestion approach. In addition, a Flag tag was fused to the N terminus of *UCP2* in the pcDNA3.1 vector using the same dual-enzyme digestion approach.

### Cell Culture and Transfection

Human embryonic kidney (HEK)-derived 293T cells were purchased from ATCC (ATCC; Manassas, VA, United States) and grown in Dulbecco’s modified Eagle medium/GlutaMaxTM medium (GIBCO, 15140–122) supplemented with 10% fetal bovine serum, 2 mM L-glutamine, and 10 U/ml Penicillin-Streptomycin at 37°C in a 5% CO_2_ incubator. The HEK cells were transiently transfected with 2 μg of plasmid DNA of wild-type or mutant CPT2 constructs using Lipofectamine 2000 according to the standard protocol from the manufacturer (Invitrogen, Grand Island, NY, United States).

### Fluorescence Microscopy

24 h after transfection, transfected HEK cells were washed three times with PBS for 3 min per wash, then fixed for 15 min in PBS containing 4% paraformaldehyde. For nuclear DNA visualization, cell samples were mounted with ProLong Gold antifade reagent containing 1:600 DAPI for 5 min while being sheltered from light. Samples were then blocked with 50% normal glycerinum for microscopic observation. All confocal images were captured using a laser scanning confocal microscope (LSM710, Zeiss). Subcellular localization of wild-type or mutant CPT2, as well as quantitation of fluorescence signal intensity, was assessed using ImageJ software.

### Immunoprecipitation Assays

To validate the interaction between UCP2 and CPT2, we performed an immunoprecipitation assay using SureBeads Protein A/G (Bio-Rad). HEK cells were transfected with wild-type or mutant CPT2 constructs as well as the UCP2 construct in different combinations. According to the standard protocols provided by the manufacturer, cells were harvested 24 h after transfection and lysed for 30 min at 4°C in lysis buffer containing 20 mM HEPES (pH 7.4), 100 mM KCl, 2 mM MgCl2, 1 mM PMSF, 1% Triton X-100, and supplemental protease inhibitors. Lysates were then centrifuged at 14,000×*g* for 10 min at 4°C, after which the supernatants were mixed with antibody (anti-flag)-conjugated magnetic beads and incubated overnight at 4°C on a rotating wheel with end-over-end mixing. Next, beads were collected from the supernatants by centrifugation and washed four times in lysis buffer. Finally, bound protein was eluted in SDS loading buffer onto a 10% acrylamide gel, resolved by SDS-PAGE, and analyzed by Western blot with the following primary antibodies: anti-flag at 1:1,000 dilution and anti-his at 1:1,000 dilution.

## Results

### Characteristics of the Patient Cohort

**FIGURE 1:**
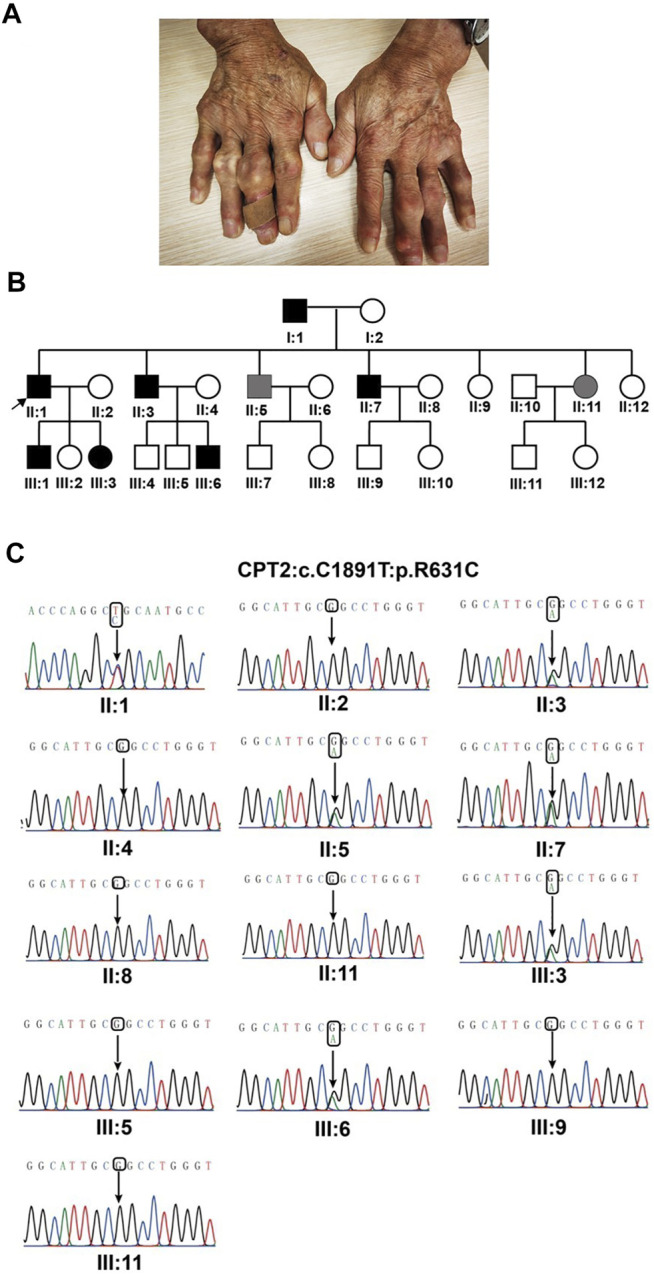
Identification of *CPT2* missense in the gout family. **(A)**. Representative photographs of the proband in gout family reveal multiple tophi, indicating long disease duration and a severe disease burden. **(B)**. Pedigree and co-segregation results. Affected individual is represented as a filled square. Normal individuals are shown as empty symbols. **(C)**. Sanger sequencing confirmed the segregation of the rare missense variant, c.1891C > T, (p.R631C).

All patients suffered acute monoarticular arthritis in the MTP1 and/or knee. Acute gouty arthritis is painful and unbearable, even occasionally accompanied by fever. All symptoms could be completely relieved within 1 week of treatment with a nonsteroidal anti-inflammatory drug and colchicines. Palpable tophi were observed in some of the patients (Figure 1A). The metabolic conditions of all patients were evaluated, and clinical data are presented in Table I. All patients have normal intelligence, and antinuclear antibody, rheumatoid factor, and HLA-B27 tests were negative for all patients.

### Detection of Rare Deleterious Variants From Proband Whole-Exome Sequencing Data

To determine the genetic etiology of gout, we performed WES on the proband (family member II:1) from a three-generation pedigree with early-onset gout. After the removal of low-quality and duplicated reads from the raw sequencing data, we identified a total of 541,954 single nucleotide variations (SNVs) and 84,415 indels using the GATK tool, of which 67,707 coding SNVs and 2,200 indels were located within splicing regions. Further application of variant filtration against variants found that at a frequency <0.001% in multiple public databases, the numbers of novel SNVs and indels potentially associated with gout were reduced to 228 and 29, respectively. To further prioritize these SNVs, we subjected the variants to functional assessment by multiple prediction tools, including SIFT, Polyphen2, MutationTaster and CADD, and retained variants predicted as deleterious by all prediction tools. Following these analyses, two SNVs in the coding regions of two gout-associated genes (*CPT2* and *ABCC4*) were retained.

**FIGURE 2:**
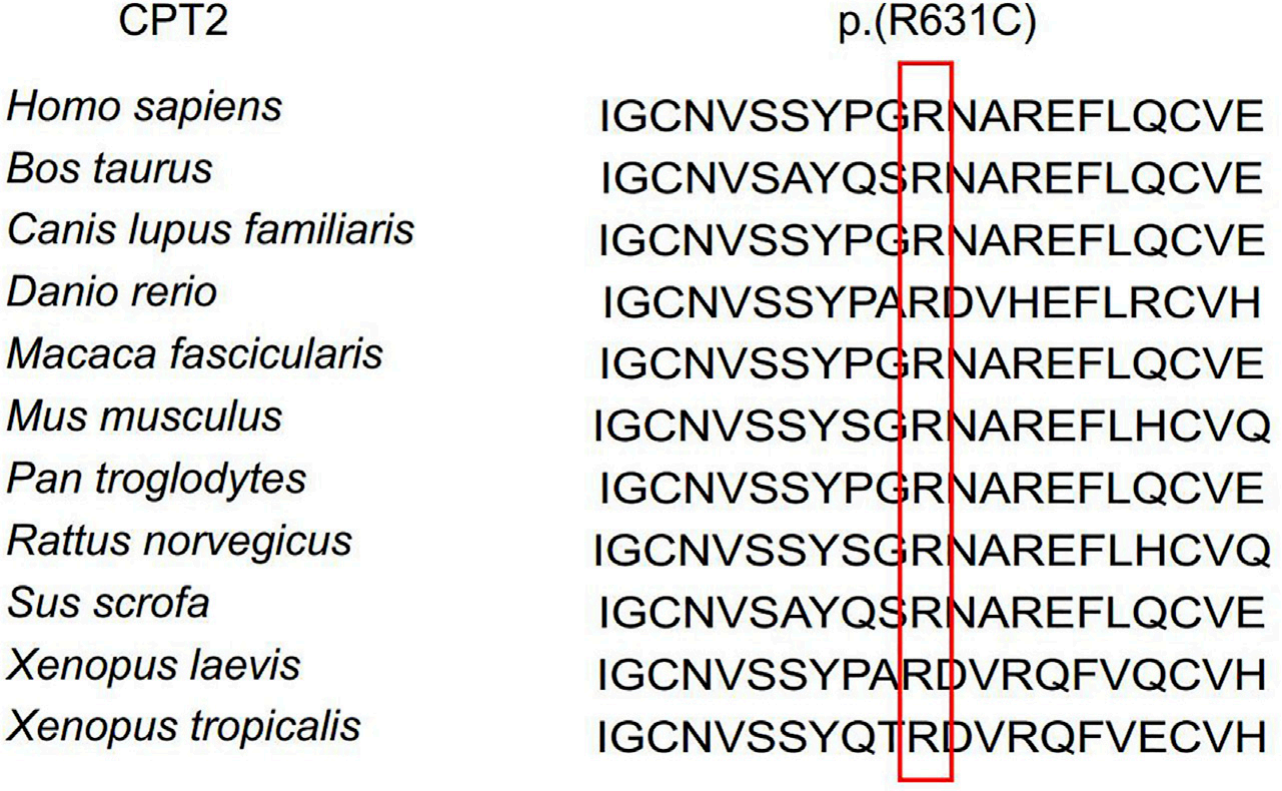
Conservation analyses. Conservation analyses of the mutated residues 631 in *CPT2* across different species.

These candidate variants were submitted for Sanger sequencing validation and co-segregation analysis in the pedigree. Following this analysis, one variant remained promising: a rare heterozygous missense variant (c.1891C > T, p.R631C) in *CPT2*. Segregation analysis confirmed that the presence of this heterozygous variant was shared by all patients, while unaffected individuals did not exhibit the nucleotide change; in other words, R631C co-segregates with the disease phenotype in this pedigree (Figures 1B,C). Additionally, *in silico* predictive algorithms for nonsynonymous variation in all prediction tools suggested strong pathogenicity for this variant based on effects on protein function. By querying against multiple public databases, we found that this variant is extremely rare (rs74315293) in gnomAD and ExAC databases, with respective allele frequencies of 0.002475% (7 in 282,810) and 0.002471% (3 in 121,398), but is not present in 1,000 genomes or ESP6500 (Table 2). The heterozygous missense variant causes an arginine (Arg) to cysteine (Cys) substitution at amino acid 631, adjacent to the C terminus. Meanwhile, multiple orthologous sequence alignment revealed that the arginine at position 631 is highly conserved among different vertebrate and invertebrate species (Figure 2). According to the standards and guidelines of ACMG, the missense variants were classified as likely pathogenic. Specifically, the interpretation for pathogenesis of variant c.1891C > T is rated as pathogenic by the ClinVar database.

**TABLE 2:** In silico analysis of the missense variant in *CPT2*

Variants	AA conservation	gnomAD (%)	ExAC (%)	1,000 genomes	ESP6500	SIFT	PolyPhen-2	CADD	Mutation Taster
p.(R631C)	Highly conserved	0.002475	0.002471	_	_	Damaging (0.004)	probably damaging (1.0)	Damaging (28.4)	Disease-causing (1.0)

Abbreviations: AA, amino acid; CADD, combined annotation dependent depletion; ExAC, exome aggregation consortium; gnomAD, genome aggregation database; PolyPhen-2, polymorphism phenotyping v2; SIFT, sorting intolerant from tolerant.

### Effects of CPT2 Variants on Protein Localization and Expression in Cells

**FIGURE 3:**
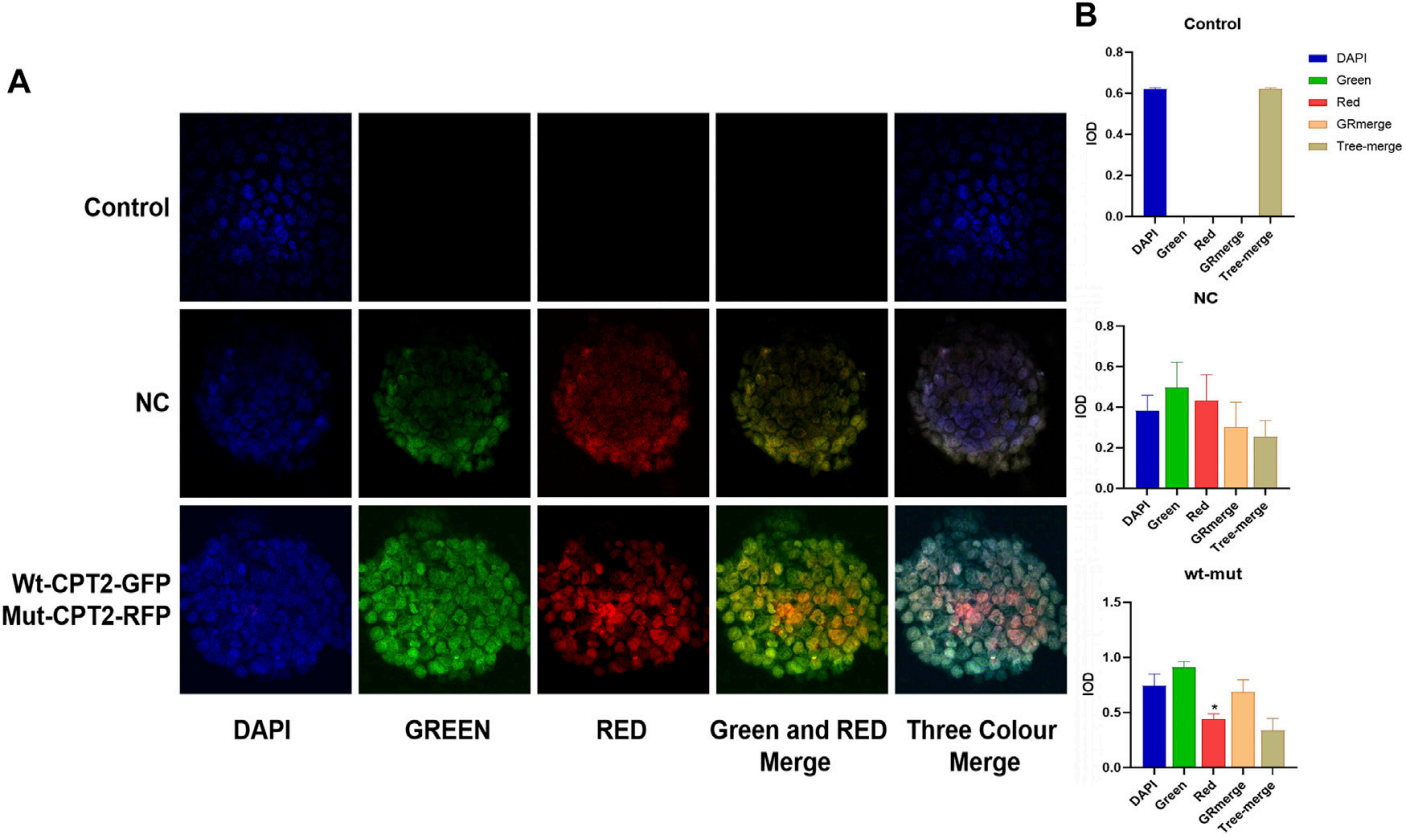
Missense variant p.R631C on the subcellular localization of CPT2 protein under confocal microscopy. **(A)**. The vectors drove the expression of the wild-type protein in association with GFP protein tag (WT-CPT2-GFP) as well as the mutant variants fusion with RFP fusion protein for identification (Mut-CPT2-GFP). The CPT2 protein was mainly localized in the nucleus, partly distributed in the cytoplasm. The fluorescence signal showed that there was no overt subcellular mislocalization of the p.R631C mutant with respect to wild-type CPT2. **(B)**. The fluorescence intensity of p.R631C mutants was obviously reduced than wild-type protein.

To assess the effects of missense variant p.R631C on the subcellular localization of CPT2 protein in affected individuals, we constructed vectors driving expression of the wild-type protein in association with a GFP protein tag (WT-CPT2-GFP), as well fusions of the mutant variant with RFP for identification (Mut-CPT2-GFP). After transfection of wild-type and mutant CPT2 constructs into HEK293 cells, cell morphology did not differ noticeably between cells overexpressing the wild-type protein or p.R631C mutant and those transfected only with GFP, RFP, or an empty vector (Figure 3A). In addition, we found that the CPT2 protein is mainly localized to the nucleus but is partially distributed in the cytoplasm. Further analysis of fluorescence signal indicated that there was no overt subcellular mislocalization of the p.R631C mutant compared to wild-type CPT2 (Figure 3A). However, the fluorescence intensity of p.R631C mutants was obviously compared to that of the wild-type protein, which could be the result of protein degradation caused by the mutations (Figure 3B).

### Functional Analyses of CPT2

**FIGURE 4:**
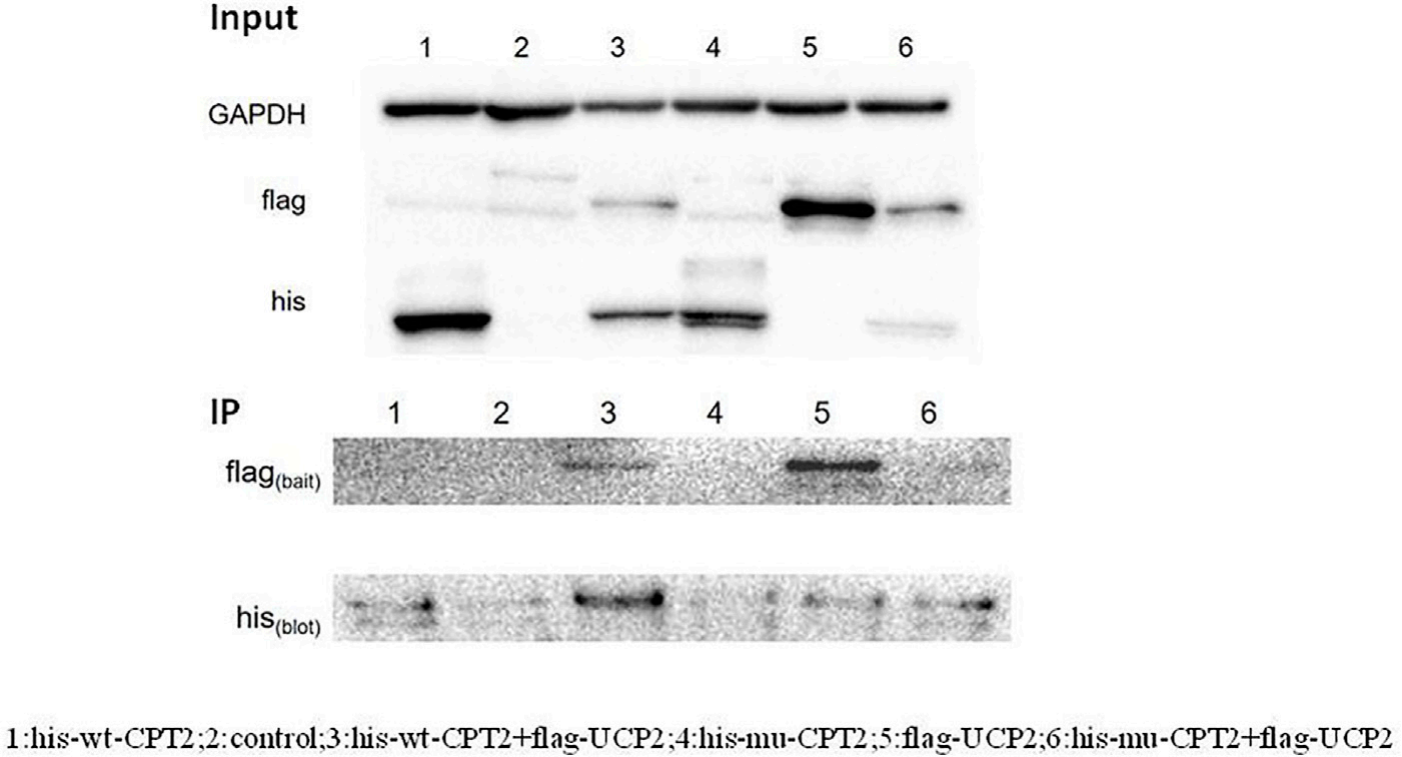
Immunoprecipitation assay of the interaction between CPT2 protein with UCP2 protein. Results of a co-immunoprecipitation assay between the CPT2-his and UCP2-fIag proteins. Immunoprecipitation with anti-FLAG beads coupled with Western blot analysis by histone antibody showed a strong physical interaction between CPT2 and UCP2. No obvious interacted signal was observed between mutant CPT2 and UCP2.

It has been previously suggested that the interaction between CPT2 and UCP2 is associated with gout or hyperuricemia. To further evaluate the possible role of CPT2 in gout, we first assessed the interaction between UCP2 and wild-type CPT2, then determined the effect of the p.R631C mutation on this interaction. Therefore, we performed an *in vitro* immunoprecipitation assay by constructing expression vectors with His tags fused to the N termini of CPT2 (His-WT-CPT2) and c.1891C > T mutants (His-mu-CPT2) and FLAG tags fused to the N terminus of UCP (Flag-UCP). These constructs were transfected into HEK293 cells alone or in combination. Immunoprecipitation with anti-FLAG beads coupled with Western blot analysis with histone antibodies showed a strong physical interaction between CPT2 and UCP2 (Figure 4). Of note, no obvious interaction signal was observed between the CPT2 mutant and UCP2, indicating that the c.1891C > T variant influences the ability of CPT2 and UCP2 to bind (Figure 4).

## Discussion

In the present study, we recruited a multi-generational pedigree with gout and comprehensively performed WES and co-segregation testing to investigate the genetic basis of gout in this family. Ultimately, this study identified a rare mutation (c.1891C > T, p.R631C) in the *CPT2* gene.

The CPT2 gene encodes carnitine palmitoyltransferase II, an enzyme that participates in fatty acid oxidation. Britton et al. distinguished CPT I and CPT II (Britton et al., 1995). Mutations in the CPT2 gene can cause Carnitine Palmitoyltransferase II Deficiency (Demaugre et al., 1991; Taroni et al., 1992; Bonnefont et al., 1996). Of note, the R631C mutation was previously reported in a patient with infantile carnitine palmitoyltransferase II deficiency (Taroni et al., 1992). Taken together, these findings highlight the genetic and clinical heterogeneity of defects in *CPT2*.

In addition to the evidence from WES and genetic analyses, this study also provides evidence from functional experiments. We constructed vectors driving expression of the wild-type protein in association with a GFP protein tag (WT-CPT2-GFP), as well as of the mutant variant fused to RFP for identification (Mut-CPT2-GFP). Although no change in localization was observed, the results showed that the fluorescence intensity of p.R631C mutants was obviously reduced compared to the wild-type protein, suggesting that the mutation leads to protein degradation.

The effect of the CPT2 mutant was further defined by studying the interaction between UCP2 and CPT2. UCP2 has been reported to be associated with serum urate concentrations, as well as with hyperuricemia (Yang et al., 2016). To assess the effects of the R631C mutation, we performed an *in vitro* immunoprecipitation assay by constructing expression vectors with His tags fused to the N termini of CPT2 (His-WT-CPT2) and c.1891C > T mutants (His-mu-CPT2) and FLAG tags fused to the N terminus of UCP (Flag-UCP). Interestingly, while our results showed a strong physical interaction between wild-type CPT2 and UCP2, no obvious interaction signal was observed between the CPT2 mutant and UCP2. These results indicate that the c.1891C > T variant influences the ability of CPT2 to bind UCP2. Taken as a whole, evidence from functional experiments further supports the pathogenicity of the R631C mutation.

Interestingly, our previous study reported an association between *SLC16A9* and gout, and *SLC16A9* is known to be associated with carnitine levels (Demirkan et al., 2015). Thus, it is reasonable to speculate that the carnitine-related pathway may play an important role in gout development.

In conclusion, this study identifies a rare *CPT2* mutation in a large Chinese pedigree with gout. Functional studies were used to determine the effect of this mutant. This study provides novel insight into the genetic etiology of gout.

## Data Availability

The data presented in the study are deposited in the SRA Bioproject repository, accession number PRJNA 776345.

## References

[B1] Bonnefont J. P. Taroni F. Cavadini P. Cepanec C. Brivet M. Saudubray J. M. (1996). Molecular Analysis of Carnitine Palmitoyltransferase II Deficiency with Hepatocardiomuscular Expression. Am. J. Hum. Genet. 58 (5), 971–978. PubMed Abstract | Google Scholar 8651281 PMC1914604

[B2] Britton C. H. Schultz R. A. Zhang B. Esser V. Foster D. W. McGarry J. D. (1995). Human Liver Mitochondrial Carnitine Palmitoyltransferase I: Characterization of its cDNA and Chromosomal Localization and Partial Analysis of the Gene. Proc. Natl. Acad. Sci. 92 (6), 1984–1988. 10.1073/pnas.92.6.1984 10.1073/pnas.92.6.1984 | Google Scholar 7892212 PMC42407

[B3] Chen X. Jin J. Wang Q. Xue H. Zhang N. Du Y. (2019). A De Novo Pathogenic CSNK1E Mutation Identified by Exome Sequencing in Family Trios with Epileptic Encephalopathy. Hum. Mutat. 40 (3), 281–287. 10.1002/humu.23690 PubMed Abstract | 10.1002/humu.23690 | Google Scholar 30488659

[B4] Demaugre F. Bonnefont J. P. Colonna M. Cepanec C. Leroux J. P. Saudubray J. M. (1991). Infantile Form of Carnitine Palmitoyltransferase II Deficiency with Hepatomuscular Symptoms and Sudden Death. Physiopathological Approach to Carnitine Palmitoyltransferase II Deficiencies. J. Clin. Invest. 87 (3), 859–864. 10.1172/jci115090 PubMed Abstract | 10.1172/jci115090 | Google Scholar 1999498 PMC329874

[B5] Demirkan A. Henneman P. Verhoeven A. Dharuri H. Amin N. van Klinken J. B. (2015). Insight in Genome-wide Association of Metabolite Quantitative Traits by Exome Sequence Analyses. Plos Genet. 11 (1), e1004835. 10.1371/journal.pgen.1004835 PubMed Abstract | 10.1371/journal.pgen.1004835 | Google Scholar 25569235 PMC4287344

[B6] Huang X. F. Sun L. Zhang C. Zhou Z. Chen H. Zhang L. (2020). Whole-Exome Sequencing Reveals a Rare Missense Variant in SLC16A9 in a Pedigree with Early-Onset Gout. Biomed. Res. Int. 2020, 4321419. 10.1155/2020/4321419 PubMed Abstract | 10.1155/2020/4321419 | Google Scholar 32090094 PMC7013288

[B7] Huang X. F. Xiang L. Cheng W. Cheng F. F. He K. W. Zhang B. W. (2018). Mutation of IPO13 Causes Recessive Ocular Coloboma, Microphthalmia, and Cataract. Exp. Mol. Med. 50 (4), 53. 10.1038/s12276-018-0079-0 10.1038/s12276-018-0079-0 | Google Scholar PMC5938035 29700284

[B8] Huang X. F. Xiang L. Fang X. L. Liu W. Q. Zhuang Y. Y. Chen Z. J. (2019). Functional Characterization of CEP250 Variant Identified in Nonsyndromic Retinitis Pigmentosa. Hum. Mutat. 40 (8), 1039–1045. 10.1002/humu.23759 PubMed Abstract | 10.1002/humu.23759 | Google Scholar 30998843

[B9] Köttgen A. Albrecht E. Teumer A. Vitart V. Krumsiek J. Hundertmark C. (2013). Genome-wide Association Analyses Identify 18 New Loci Associated with Serum Urate Concentrations. Nat. Genet. 45 (2), 145–154. 10.1038/ng.2500 PubMed Abstract | 10.1038/ng.2500 | Google Scholar 23263486 PMC3663712

[B10] Li C. Li Z. Liu S. Wang C. Han L. Cui L. (2015). Genome-wide Association Analysis Identifies Three New Risk Loci for Gout Arthritis in Han Chinese. Nat. Commun. 6, 7041. 10.1038/ncomms8041 PubMed Abstract | 10.1038/ncomms8041 | Google Scholar 25967671 PMC4479022

[B11] Li H. Durbin R. (2010). Fast and Accurate Long-Read Alignment with Burrows-Wheeler Transform. Bioinformatics 26 (5), 589–595. 10.1093/bioinformatics/btp698 PubMed Abstract | 10.1093/bioinformatics/btp698 | Google Scholar 20080505 PMC2828108

[B12] Li J. Jiang Y. Wang T. Chen H. Xie Q. Shao Q. (2015). mirTrios: an Integrated Pipeline for Detection of De Novo and Rare Inherited Mutations from Trios-Based Next-Generation Sequencing. J. Med. Genet. 52 (4), 275–281. 10.1136/jmedgenet-2014-102656 PubMed Abstract | 10.1136/jmedgenet-2014-102656 | Google Scholar 25596308

[B13] Liu Q. Chen C. Shen E. Zhao F. Sun Z. Wu J. (2012). Detection, Annotation and Visualization of Alternative Splicing from RNA-Seq Data with SplicingViewer. Genomics 99 (3), 178–182. 10.1016/j.ygeno.2011.12.003 PubMed Abstract | 10.1016/j.ygeno.2011.12.003 | Google Scholar 22226708

[B14] Liu Z. Li Z. Zhi X. Du Y. Lin Z. Wu J. (2018). Identification of De Novo DNMT3A Mutations that Cause West Syndrome by Using Whole-Exome Sequencing. Mol. Neurobiol. 55 (3), 2483–2493. 10.1007/s12035-017-0483-9 PubMed Abstract | 10.1007/s12035-017-0483-9 | Google Scholar 28386848

[B15] Major T. J. Dalbeth N. Stahl E. A. Merriman T. R. (2018). An Update on the Genetics of Hyperuricaemia and Gout. Nat. Rev. Rheumatol. 14 (6), 341–353. 10.1038/s41584-018-0004-x PubMed Abstract | 10.1038/s41584-018-0004-x | Google Scholar 29740155

[B16] Matsuo H. Yamamoto K. Nakaoka H. Nakayama A. Sakiyama M. Chiba T. (2016). Genome-wide Association Study of Clinically Defined Gout Identifies Multiple Risk Loci and its Association with Clinical Subtypes. Ann. Rheum. Dis. 75 (4), 652–659. 10.1136/annrheumdis-2014-206191 PubMed Abstract | 10.1136/annrheumdis-2014-206191 | Google Scholar 25646370 PMC4819613

[B17] Nakayama A. Nakaoka H. Yamamoto K. Sakiyama M. Shaukat A. Toyoda Y. (2017). GWAS of Clinically Defined Gout and Subtypes Identifies Multiple Susceptibility Loci that Include Urate Transporter Genes. Ann. Rheum. Dis. 76 (5), 869–877. 10.1136/annrheumdis-2016-209632 PubMed Abstract | 10.1136/annrheumdis-2016-209632 | Google Scholar 27899376 PMC5530361

[B18] Neogi T. Jansen T. L. T. A. Dalbeth N. Fransen J. Schumacher H. R. Berendsen D. (20152015). 2015 Gout Classification Criteria: An American College of Rheumatology/European League against Rheumatism Collaborative Initiative. Arthritis Rheumatol. 67 (10), 2557–2568. 10.1002/art.39254 PubMed Abstract | 10.1002/art.39254 | Google Scholar PMC4566153 26352873

[B19] Phipps-Green A. J. Merriman M. E. Topless R. Altaf S. Montgomery G. W. Franklin C. (2016). Twenty-eight Loci that Influence Serum Urate Levels: Analysis of Association with Gout. Ann. Rheum. Dis. 75 (1), 124–130. 10.1136/annrheumdis-2014-205877 PubMed Abstract | 10.1136/annrheumdis-2014-205877 | Google Scholar 25187157

[B20] Richette P. Bardin T. (2010). Gout. The Lancet 375 (9711), 318–328. 10.1016/s0140-6736(09)60883-7 10.1016/s0140-6736(09)60883-7 | Google Scholar 19692116

[B21] Sulem P. Gudbjartsson D. F. Walters G. B. Helgadottir H. T. Helgason A. Gudjonsson S. A. (2011). Identification of Low-Frequency Variants Associated with Gout and Serum Uric Acid Levels. Nat. Genet. 43 (11), 1127–1130. 10.1038/ng.972 PubMed Abstract | 10.1038/ng.972 | Google Scholar 21983786

[B22] Taroni F. Verderio E. Fiorucci S. Cavadini P. Finocchiaro G. Uziel G. (1992). Molecular Characterization of Inherited Carnitine Palmitoyltransferase II Deficiency. Proc. Natl. Acad. Sci. 89 (18), 8429–8433. 10.1073/pnas.89.18.8429 PubMed Abstract | 10.1073/pnas.89.18.8429 | Google Scholar 1528846 PMC49933

[B23] Yang L. Dong Z. Zhou J. Ma Y. Pu W. Zhao D. (2016). Common UCP2 Variants Contribute to Serum Urate Concentrations and the Risk of Hyperuricemia. Sci. Rep. 6, 27279. 10.1038/srep27279 PubMed Abstract | 10.1038/srep27279 | Google Scholar 27273589 PMC4897637

