# Corrigendum: Whole-exome sequencing identifies a novel CPT2 mutation in a pedigree with gout

**DOI:** 10.3389/fcell.2022.988348

**Published:** 2022-08-04

**Authors:** Yong Guo, Jing Jin, Zhenni Zhou, Yihui Chen, Li Sun, Chunwu Zhang, Xiaoru Xia

**Affiliations:** ^1^ Department of Urology, The First Affiliated Hospital of Wenzhou Medical University, Wenzhou, China; ^2^ Zhejiang Center for Clinical Laboratory, Zhejiang Provincial People’s Hospital, Affiliated People’s Hospital, Hangzhou Medical College, Hangzhou, China; ^3^ Department of Internal Medicine, Yueqing People’s Hospital, Yueqing, China; ^4^ Wenzhou Medical University, Wenzhou, China; ^5^ Department of Rheumatology, The First Affiliated Hospital of Wenzhou Medical University, Wenzhou, China; ^6^ Department of Injury Orthopaedics, The First Affiliated Hospital of Wenzhou Medical University, Wenzhou, China

**Keywords:** whole-exome sequencing, novel mutation, gout, CPT2 gene, pedigree

In the published article, there was an error in affiliations 1 and 6. Instead of “The First Affiliated Hospital of Wenzhou University”, it should be “The First Affiliated Hospital of Wenzhou Medical University”.

The authors apologize for this error and state that this does not change the scientific conclusions of the article in any way. The original article has been updated.

